# A new recombineering system for precise genome-editing in *Shewanella oneidensis* strain MR-1 using single-stranded oligonucleotides

**DOI:** 10.1038/s41598-018-37025-4

**Published:** 2019-01-10

**Authors:** Anna D. Corts, Lynn C. Thomason, Ryan T. Gill, Jeffrey A. Gralnick

**Affiliations:** 1 BioTechnology Institute and Department of Plant and Microbial Biology, University of Minnesota-Twin Cities, St. Paul, MN 55108 USA; 2 RNA Biology Laboratory, Basic Science Program, Leidos Biomedical Inc., Frederick National Laboratory for Cancer Research, Frederick, MD 21702 USA; 3 Department of Chemical and Biological Engineering, University of Colorado-Boulder, Boulder, CO 80303 USA

## Abstract

*Shewanella oneidensis* MR-1 is an invaluable host for the discovery and engineering of pathways important for bioremediation of toxic and radioactive metals and understanding extracellular electron transfer. However, genetic manipulation is challenging due to the lack of genetic tools. Previously, the only reliable method used for introducing DNA into *Shewanella spp*. at high efficiency was bacterial conjugation, enabling transposon mutagenesis and targeted knockouts using suicide vectors for gene disruptions. Here, we describe development of a robust and simple electroporation method in *S*. *oneidensis* that allows an efficiency of ~4.0 x 10^6^ transformants/µg DNA. High transformation efficiency is maintained when cells are frozen for long term storage. In addition, we report a new prophage-mediated genome engineering (recombineering) system using a λ Red Beta homolog from *Shewanella* sp. W3-18-1. By targeting two different chromosomal alleles, we demonstrate its application for precise genome editing using single strand DNA oligonucleotides and show that an efficiency of ~5% recombinants among total cells can be obtained. This is the first effective and simple strategy for recombination with markerless mutations in *S*. *oneidensis*. Continued development of this recombinant technology will advance high-throughput and genome modification efforts to engineer and investigate *S*. *oneidensis* and other environmental bacteria.

## Introduction

Bacteria from the genus *Shewanella* are aquatic and facultative anaerobic microorganisms of important interest because of their wide respiratory capabilities^1,2^, ranging from using metals such as Cr(VI)^3,4^ to electrodes^5–8^ to solvents like dimethyl sulfoxide (DMSO)^9,10^ as electron acceptors. As a result, *Shewanella* show great potential for remediation of various environmental pollutants and electrical current-generation for use in applications such as waste water treatment. Although most members of this group are easily cultured in the laboratory, a major barrier preventing metabolic engineering of this organism has been a lack of methodologies for DNA transformation and tools for precise, large-scale genome engineering. To date, the most reliable method used for introducing DNA into *Shewanella* spp. has been bacterial conjugation, a somewhat tedious and time-consuming technique when compared to electroporation-based methodologies. Transposon mutagenesis and targeted knockouts by suicide vectors have been extensively used for gene manipulation, however these tools are typically used in gene disruption applications and are inadequate for metabolic engineering purposes.

*Shewanella oneidensis* strain MR-1 is a widely used *Shewanella* strain but displays a low efficiency of electrotransformation for heterologous plasmids derived from different bacterial species, due to its native restriction-modification system^11^. Since the first attempt of electrotransformation in *S*. *oneidensis* in 1994^12^, only a few studies have used electroporation as a method to transfer DNA in *Shewanella*^11,13–16^, and no robust transformation protocol has been established to date. A recent study in the model organism *E*. *coli* showed that making electrocompetent cells and performing electroporation at room temperature^17^ improves DNA transformation up to 10-fold. Although standard procedure is to keep cells on ice when making electrocompetent cells and during electroporation^18^, we found that preparation of electrocompetent cells at room temperature also increases transformation efficiency in *S*. *oneidensis*. Additionally, we optimized a number of other transformation parameters including DNA concentration, cell quantity, growth phase, electroporation conditions and cell competence after freezing at −80 °C. We found that using cells grown to late exponential phase increases electroporation efficiency by nearly 400-fold compared to those from early exponential phase, as conventional methods describe. This surprising finding allowed us to make electrocompetent cells by simply using overnight cultures which, when frozen at −80 °C, still maintain the same high transformation efficiency.

Developing this high efficiency method for DNA uptake in *S*. *oneidensis* has enabled us to introduce single-stranded DNA oligonucleotides (oligos) to perform *in vivo* homologous recombination–mediated genetic engineering, known as recombineering, a powerful tool for precise DNA editing developed in *E*. *coli*. Recombineering enables efficient and rapid *in vivo* construction of mutant alleles by taking advantage of homologous recombination mediated by expression of bacteriophage proteins, such as the Red system from phage λ and the RecET system from the Rac prophage. Linear DNA fragments, either double-stranded DNA (dsDNA) or single-stranded DNA (ssDNA) can be designed with short homology sequences, as short as 50 base pairs (bp), to a genomic target allowing accurate insertion, deletion or alteration of any DNA sequence without relying on conveniently located restriction sites^19–21^. The λ Red system includes three proteins: Exo, Beta, and Gam. Exo is a dsDNA exonuclease, which degrades DNA in the 5′ to 3′ direction creating 3′ ssDNA overhangs; the recombinase Beta is a single-strand annealing protein, which binds to these ssDNA overhangs and pairs them with complementary ssDNA targets^22^. Exo and Beta are functionally equivalent although not related at the sequence level to RecE and RecT, respectively. The Gam protein inhibits the RecBCD and SbcCD nuclease activities in the host, protecting the exogenous DNA from being degraded^23^. The Rac prophage does not encode a known analogue of Gam, but a study showed it can be combined with RecET^24^. Recombineering with dsDNA requires the presence of both exonuclease and recombinase^25^, but ssDNA recombineering only requires the expression of the recombinase^26^.

**Figure 1:**
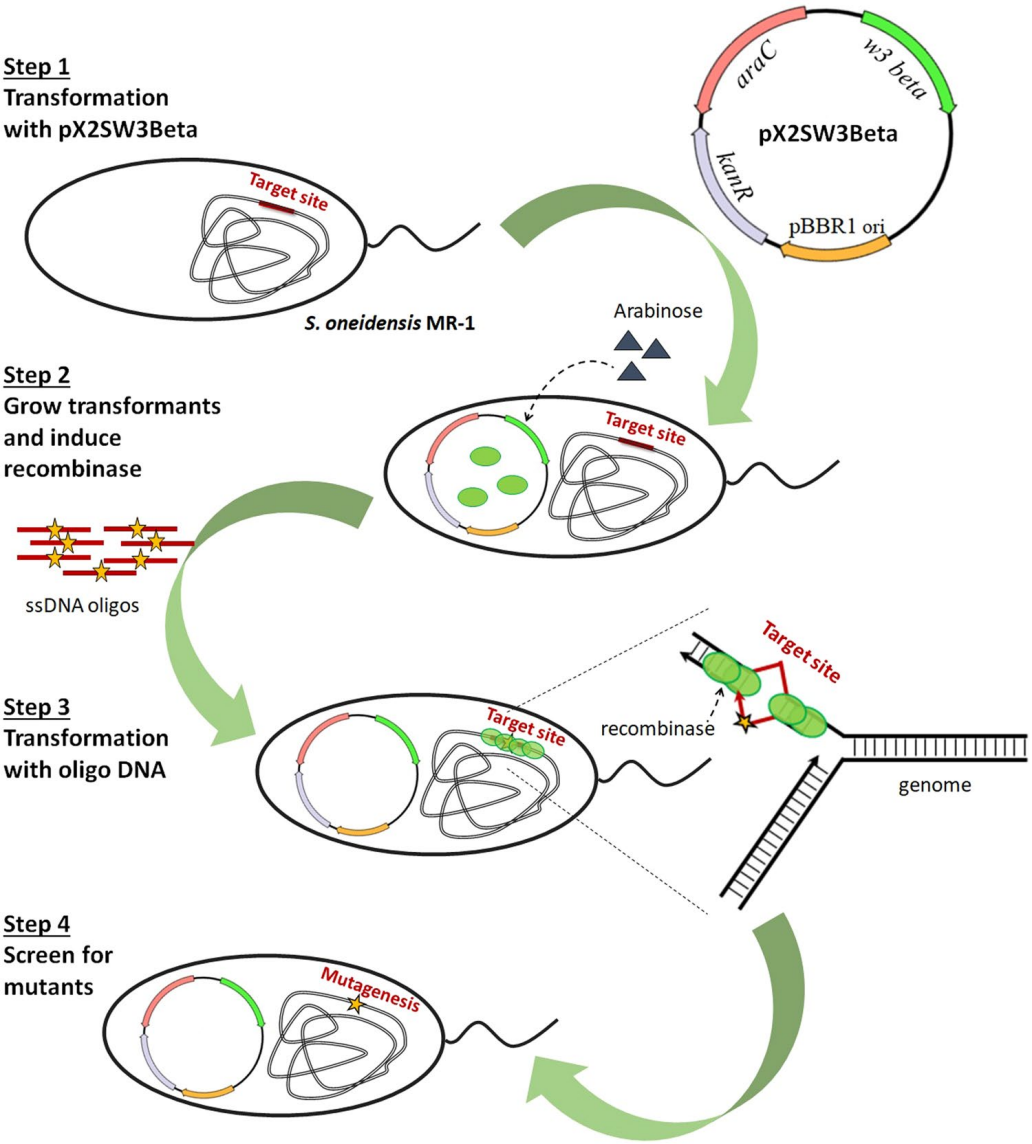
Representation of the stepwise approach used for recombineering in *S*. *oneidensis*. The system consists of one plasmid, pX2SW3Beta, expressing the recombinase (*w3 Beta*) under the inducible arabinose promoter which is regulated by the arabinose repressor (*araC*). The plasmid is introduced into host cells by electrotransformation, followed by plating on LB + Km and growth at 30 °C. The recombinase resident in the resulting strain is induced with arabinose and the cells are made electrocompetent. ssDNA oligos are transferred by electroporation to generate targeted mutations. After recovery, the cells are plated on LB + Km and incubated at 30 °C. Mutants are then screened by PCR and the process can be repeated.

Adapting recombineering for a new species is challenging, however, because the λ Red and RecET systems do not necessarily maintain high efficiency across different bacteria^27–29^, suggesting a dependence on host-specific machinery^25,30^. Since recombineering was first applied in *E*. *coli*^31,32^, a few other phage homologous recombination systems have been found to promote recombination in *Pseudomonas*, *Vibrio*, *Lactobacillus*, *Mycobacteria*, *Photorhabdus* and *Staphylococcus*^25,33–40^, and we have explored whether this technology can be established in *S*. *oneidensis*. To maximize the recombination efficiency, we identified λ Red recombinase homologs in *Shewanella* species and have tested a system from *Shewanella* sp. W3-18-1^41^. Here, we demonstrate the use of *Shewanella* sp. W3-18-1 recombinase, which shares 55% identity to the λ Red Beta recombinase from *E*. *coli* phage λ, for targeted mutagenesis using ssDNA in *S*. *oneidensis*. This recombinase is also active in wild type *E*. *coli* MG1655, achieving higher levels of recombination than RecT and comparable to λ Red Beta. This recombineering process, outlined in Fig. 1, can increase the ease with which complex genome engineering efforts are completed in *S*. *oneidensis*. In contrast to other mutagenesis methods, this technology allows precise editing at the single nucleotide level in a few days.

## Results

### Room temperature enhances electrotransformation efficiency in *S*. *oneidensis*

We tested transformation of plasmids pACYC’ (a modified version of pACYC184^13^, 2,703 bp) with chloramphenicol (Cm) resistance and pBTBX2^42^ (3,831 bp) with kanamycin (Km) resistance, by making electrocompetent cells at room temperature (RT). Each plasmid was purified from the methylation-proficient *E*. *coli* UQ950 and from a methylation-minus *E*. *coli* (GM1674 or GM2163). The latter contains a mutation in the DNA-cytosine methyltransferase (*dcm*^−6^) that prevents methylation of cytosine residues, which is useful for plasmid purification and transformation into other bacteria because of host modification-dependent restriction systems^43^.

**Figure 2:**
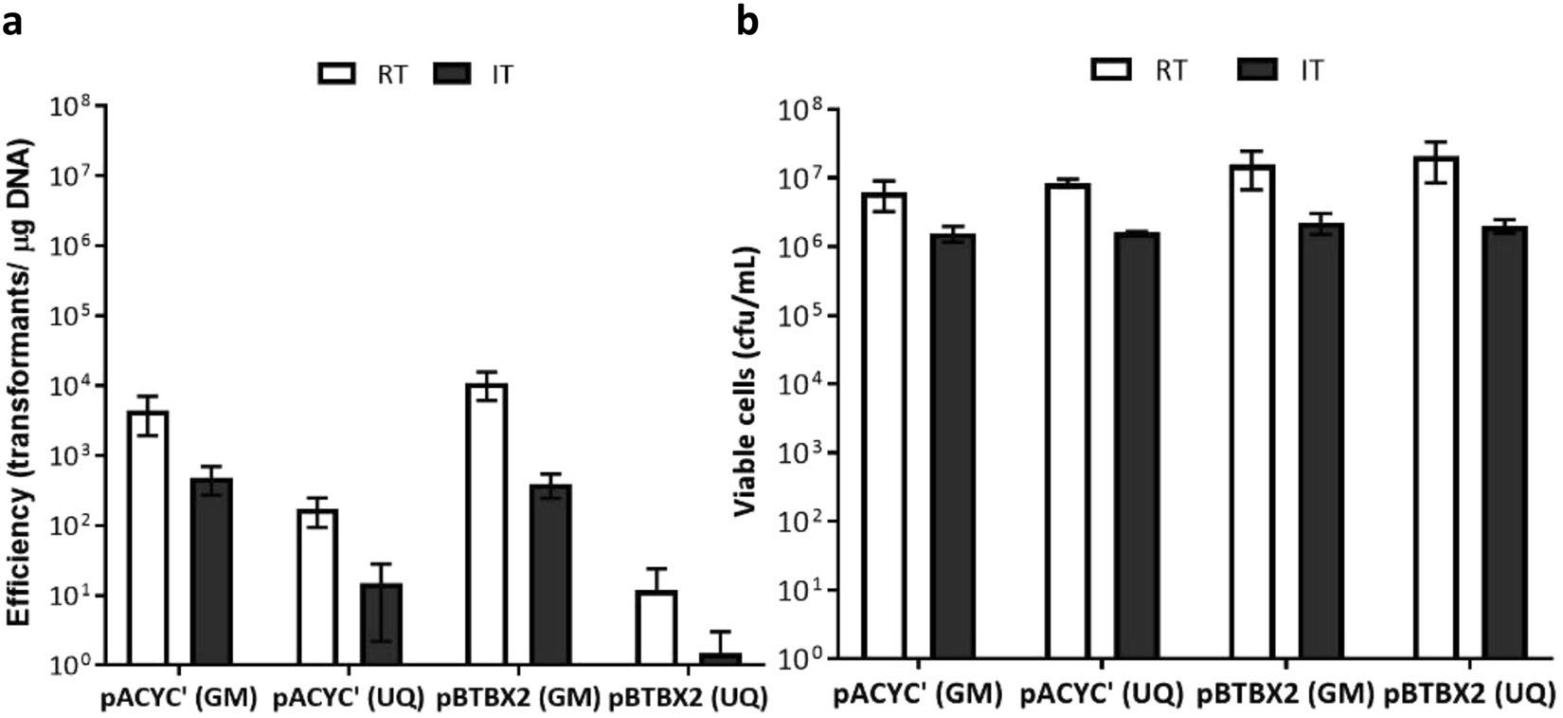
Electrotransformation efficiency and cell viability at room temperature (RT) and ice temperature (IT). (**a**) Transformation efficiency. (**b**) Cell viability. The error bars represent standard error from three independent experiments. Cells were transformed with 250 ng of plasmid pACYC’ or pBTBX2 purified from a methylation-minus (GM) or -proficient (UQ) *E*. *coli*.

No matter the plasmid type or source, the electroporation efficiency was consistently higher for electrocompetent cells prepared at room temperature (Figs 2a and S1a). In addition, transformation efficiencies were higher when plasmids were purified from the methylation-minus *E*. *coli*, as expected.

We observed that cell viability decreased between 4- to 10-fold when making electrocompetent cells on ice (Fig. 2b), suggesting cell lysis, as was observed for *E*. *coli*^17^. Further analysis by colony PCR and agarose gel electrophoresis of transformants obtained at room temperature indicated successful transformation (Fig. S1b). In parallel experiments, mock transformations without plasmid produced no colonies on selective agar plates.

### Development of an efficient electroporation method in *S*. *oneidensis*

At room temperature the absolute number of transformants increased, but the efficiency was still low. Thus, it was necessary to evaluate other electroporation factors to achieve higher efficiency. We optimized these parameters using the larger plasmid pBTBX2 purified from the methylation-minus *E*. *coli* and further verified our observations using pBTBX2 purified from *E*. *coli* UQ950 and wild-type *S*. *oneidensis*.

**Figure 3:**
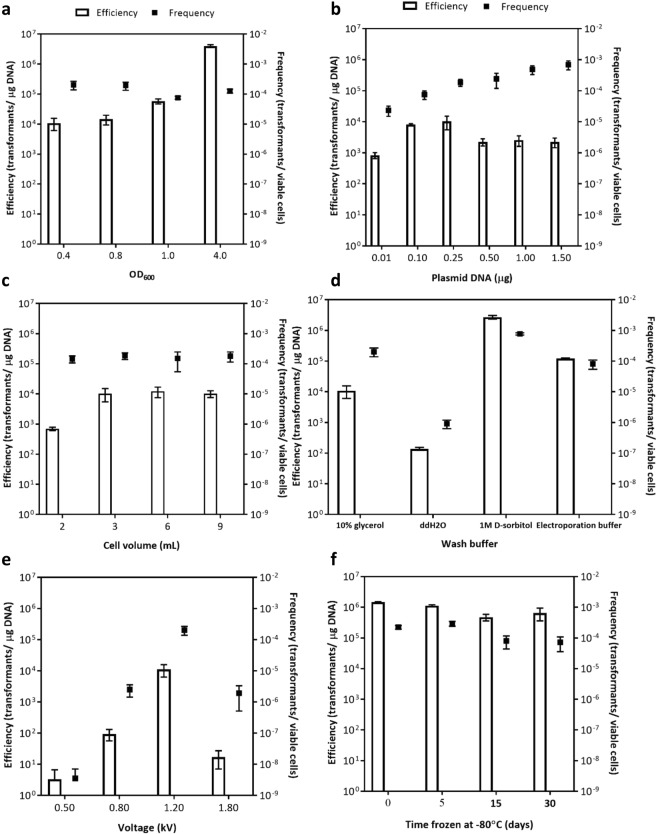
Effects of various parameters on *S*. *oneidensis* transformation using plasmid pBTBX2 purified from methylation-minus *E*. *coli* GM2163. (**a**) Cell density. (**b**) Plasmid DNA amount. (**c**) Cell volume. (**d**) Wash buffer. (**e**) Voltage. (**f**) Time frozen in 10% glycerol at −80 °C (one mL cells was used). The error bars represent standard error from three independent experiments. Three mL cells were transformed with 250 ng of plasmid DNA, unless otherwise specified.

Cell density and growth phase are essential parameters that can be manipulated to enhance transformation efficiency. We investigated the impact of a range of cell densities from early exponential phase to late exponential phase. Notably, higher density resulted in a significant increase in transformation efficiency, up to ~4 × 10^6^ transformants/μg DNA using overnight cultures at late exponential phase, nearly 400-fold more transformants than at early exponential phase (Fig. 3a). These results are in contrast to *E*. *coli* electrotransformation^17,18,44^, although in agreement with transformation studies in *P*. *aureginosa*^45^. We observed a similar increase in transformants using plasmid purified from *E*. *coli* UQ950, ~450-fold higher efficiency compared to results with early exponential phase cells. When using plasmid DNA purified from *S*. *oneidensis* (Fig. S2), the efficiency increased ~40-fold, reaching that of wild-type *E*. *coli* MG1655 (~1 × 10^4^ transformants/µg DNA in our lab, using plasmid pBTBX2 purified from *E*. *coli* UQ950), presumably because of the restriction system active in that strain.

Various amounts of plasmid DNA (0.01–1.5 µg) were used to determine the optimal concentration for transformation. As we expected, a linear relationship between plasmid DNA quantity and number of transformants was observed. Specifically, 0.1 µg was sufficient for high transformation efficiency (Fig. 3b), suggesting that saturation may occur beyond this concentration. Frequency, however, resulted in a slight proportional relationship because of a slight decrease in cell viability due to dilution of cells as more DNA is added. Cell number is another parameter that can be manipulated to increase transformation efficiency. Usually, large volumes of cells are used for *E*. *coli* transformation^18^, thus we investigated the effect of using higher quantities of cells by varying the cell volume used per electroporation reaction, from 1 to 9 mL (Fig. 3c). When using one mL cells, the total viable cells surviving electroporation was only ~1.5 × 10^4^ cfu/mL and no transformants were obtained, while in three mL, ~1.6 × 10^7^ cfu/mL viable cells remained after electroporation, resulting in the highest efficiency. Increasing the cell volume to 6 and 9 mL slightly increased the total viable cells but no significant difference in the number of transformants was observed. Frequency always remained constant, ~2 × 10^−4^ transformants/ viable cell, no matter the number of cells surviving.

Various electroporation parameters had been used in earlier *S*. *oneidensis* transformation attempts^11–15^. We found that washing the cells with dH_2_O decreased both the transformation efficiency and frequency, ~80- and ~200-fold, respectively, compared to results using 10% glycerol. In contrast, 1 M sorbitol and electroporation buffer^46^ significantly increased the number of viable cells surviving electroporation (Fig. S3), resulting in ~250- and ~10-fold higher efficiency, respectively, than the 10% glycerol wash and a similar frequency (Fig. 3d). The transformation efficiency increased proportionally to the voltage applied, achieving an optimum before decreasing at high voltage. Specifically, 12 kV/cm gave the best results, ~1 × 10^4^ transformants/μg DNA (Fig. 3e) with a time constant of ~5 ms, which results in a 100- to 3,300-fold increase compared to the other voltages applied.

Using the optimized procedure summarized in Table S4, we examined whether storage at −80 °C of late exponential phase electrocompetent cells affected transformation yields. Cells frozen for 30 days maintained as high a transformation efficiency as freshly prepared cells without loss of competence when stored in 10% glycerol (Fig. 3f) or 1 M sorbitol (Fig. S4).

### Recombineering in *S*. *oneidensis* with ssDNA oligos

The significant increase in plasmid transformation efficiency was not our end goal, as we wished to determine whether precise genome editing using recombineering was possible in *S*. *oneidensis*. For this purpose, we first evaluated oligo recombineering mediated by λ Red Beta and RecT. We expressed the recombinases under the arabinose inducible promoter pBAD from the pBTBX2 plasmid, and targeted the *E*. *coli lacZ* gene integrated in a single copy on the *S*. *oneidensis* chromosome. Although in our plasmid transformation optimization we obtained higher efficiencies using late exponential phase cells, actively replicating chromosomes are fundamental when performing recombineering as these recombinases exploit replication forks^47^, thus we used cells at early exponential phase. Additionally, sorbitol was used as wash buffer since it gave the best results when using early exponential phase cells.

**Figure 4:**
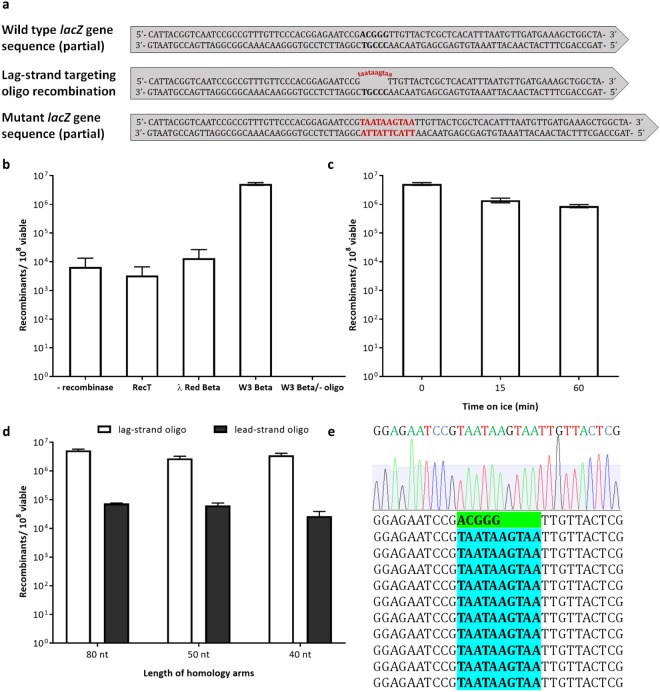
Recombineering of *lacZ* in *S*. *oneidensis* using W3 Beta recombinase. (**a**) Disruption of chromosomal *lacZ* in *S*. *oneidensis*. Sequence corresponding to the site of the mutation is shown in bold. A recombinogenic oligonucleotide was utilized to introduce ten consecutive base pair changes, which resulted in 10 bp mutations coupled with a frameshift, shown in red. (**b**) Activity of different recombinases when expressed exogenously in *S*. *oneidensis* compared to recombinase-independent (pBTBX2 empty plasmid) recombination and a mock transformation control lacking mutagenic oligonucleotide (80 nt homology arms, lag-strand oligo). (**c**) Effect of temperature when making electrocompetent cells on recombineering. Cells were prepared at room temperature or kept on ice (15 or 60 min before washing) followed by the conventional preparation on ice prior to electroporation (80 nt homology arms, lag-strand oligo). (**d**) Homology arm length of the oligo and targeting strand effect on recombineering. The number of recombinants was calculated based on the *lacZ-* colonies on the X-gal plate. The error bars represent standard error from three independent experiments. (**e**) Sequence confirmation of recombinants identified by white/blue screening, which revealed the mutation of the DNA sequence ACGGG to TAATAAGTAA for all samples, as expected (10 mutants are shown here).

Previous studies in *E*. *coli* show that most single base pair mistmatches as well as insertions up to three nucleotides are corrected by the methyl-directed mismatch repair (MMR) system present in the cell^21,48,49^. On this basis, we used synthetic ssDNA oligos containing a 10 nucleotide (nt) mutation to disrupt *lacZ* (Fig. 4a), which heterology evades the MMR and causes a mutant *lacZ-* phenotype that can be identified by white/blue screening^50^. Unexpectedly, λ Red Beta caused a significant growth defect when expressed in *S*. *oneidensis* (Fig. S5) at low levels of induction and was not functional in *S*. *oneidensis*. The number of recombinants obtained when expressing λ Red Beta was marginally above the minus-recombinase control (Fig. 4b), ~1 × 10^4^ recombinants in 10^8^ viable cells, suggesting that a low level of recombination occurs by a recombinase-independent process. Although RecT was not detrimental to the growth of *S*. *oneidensis* (Fig. S5), this recombinase was also not functional (Fig. 4b). Thus, we searched for a new recombinase from a native *Shewanella* phage and identified a potential λ Red Beta homolog (Fig. S6) in *Shewanella* sp. W3-18-1 (NCBI accession WP_011788511, locus Sputw3181_1153, annotated herein as W3 Beta), which did not adversely affect cell growth when induced (Fig. S5) and was highly efficient for oligo recombineering (Fig. 4b), obtaining ~5 × 10^6^ recombinants in 10^8^ viable cells. No recombinants were observed in control experiments lacking oligo in the system.

Tu and colleagues^17^ found that λ Red recombineering of a plasmid with dsDNA in *E*. *coli* was improved ~3-fold when the electrocompetent cells were prepared on ice and shifted to room temperature for three minutes prior to electroporation. We sought to test recombineering with W3 Beta in *S*. *oneidensis* by preparing the electrocompetent cells at different temperatures. We found that room temperature prepared cells resulted in ~6-fold more recombinants when compared to preparing cells on ice, even if we allowed transient swelling of the cells for three minutes at room temperature (Fig. 4c). As in our plasmid transformations, we observed a decrease in cell viability when cells were prepared on ice. Similarly to *E*. *coli*^21,49^, most colonies were still *lacZ*^+^, sectored blue and white, indicating the segregation of recombinant chromosomes in the original recombinant cell. Sectored colonies were streaked for isolated *lacZ*^-^ pure colonies, followed by PCR amplification of *lacZ* and sequencing of 27 colonies from these different temperature tests, which confirmed the mutations introduced (Fig. 4e).

It has been shown in *E*. *coli* that 40 nt of homology upstream and downstream of the target site is sufficient to achieve high efficiency of oligo recombineering^21^. We tested the system using oligos containing different length of homology arms ranging from 40 nt to 80 nt to find the minimum optimal length in *S*. *oneidensis*. Although 80 nt resulted in higher efficiency, generating up to ~5% recombinants among treated cells, the difference was marginal, as we obtained ~3% recombinants with the shortest 40 nt homology length (Fig. 4d).

A clear bias for oligo annealing to the lagging strand versus the leading strand has been demonstrated in *E*. *coli*^25,26,47,49^. We sought to evaluate this bias with W3 Beta in *S*. *oneidensis*. All the oligos tested corresponding in sequence to the leading strand and thus complementary to the lagging strand (lag-strand oligo) generated higher numbers of recombinants than the oligos complementary to the leading strand (lead-strand oligo) (Fig. 4d).

**Figure 5:**
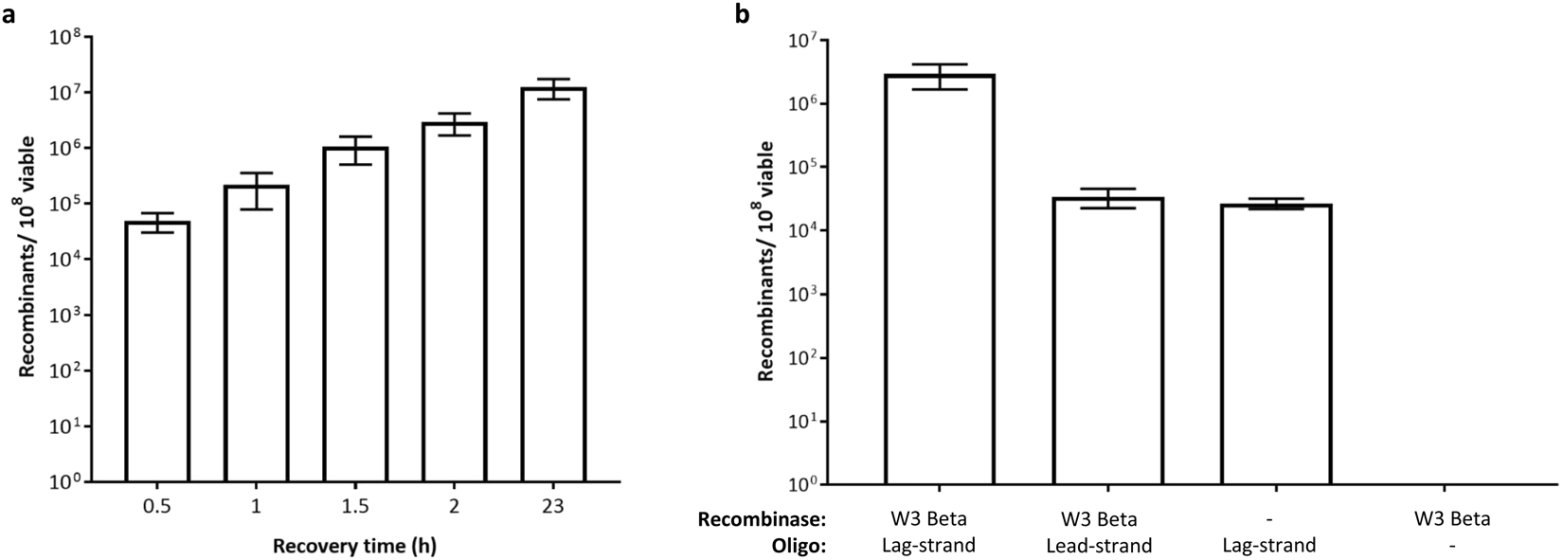
Recombineering of *rpsL* in *S*. *oneidensis* using W3 Beta recombinase. (**a**) Effect of recovery time on recombineering of the *rpsL*K43R oligo (40 nt homology arms, lag-strand oligo). (**b**) Targeting strand effect on recombineering compared to recombinase-independent and mock transformation lacking mutagenic oligonucleotide controls (40 nt homology arms oligo, 2 h recovery). The number of recombinants was calculated based on the SpectR colonies. The error bars represent standard error from three independent experiments.

We further demonstrate oligo recombineering targeting the *rpsL* gene in the chromosome of *S*. *oneidensis*. Similar to Swingle and colleagues^36,51^, the oligos were designed to introduce 4 bp changes containing the K43R (AAA- > CGG) mutation, which confers spectinomycin resistance (SpectR), and a synonymous mutation in P42 (CCT- > CCA) to evade MMR (Fig. S7a,b). Short homology arms of 40 nt were used as it resulted in sufficient length for high efficiency in our previous results targeting *lacZ*. The frequency of recombination was determined by the number of colonies that grew on streptomycin-containing LB agar, thus a longer outgrowth was necessary to allow segregation of the recombinant allele before selection (Fig. 5a). Again, a difference in efficiency was observed based on the target strandedness, specifically ~3 × 10^6^ and ~3 × 10^4^ recombinants were obtained when targeting the lagging and leading strand, respectively (Fig. 5b). The percentage of recombinants among viable cells was lower than when editing *lacZ*, ~1% targeting the lagging strand, due to the increased recovery step to obtain pure *rpsL*K43R genotypes^52^. Without the addition of the recombinase in the cells, ~3 × 10^4^ recombinants were obtained by targeting the lagging strand, in agreement with our *lacZ* editing experiments. Ten mutants each from the + and − recombinase experiments were screened by colony PCR with a reverse primer specific to the mutation introduced, which revealed that all recombinants contained the correct 4 bp change (Fig. S7c). Sequencing further confirmed the success of the experiment (Fig. S7d).

Surprisingly, W3 Beta was also functional in *E*. *coli* at an efficiency comparable to λ Red Beta. In fact, we observed that preparing electrocompetent cells and performing electroporation at room temperature also increased the number of recombinants ~5-fold when using λ Red Beta and W3 Beta in *E*. *coli* (Fig. S8a), in contrast to the dsDNA plasmid recombineering previous findings^17^. Accomplishment of the DNA change introduced by W3 Beta was confirmed by colony PCR of 14 mutants with a reverse primer specific to the mutation and sequencing of 10 colonies (Fig. S8b,c).

## Discussion

The ability to transfer exogenous DNA into cells is essential for genetic engineering of microorganisms. Although conjugation has been widely used as the means to transfer plasmid DNA in bacteria, electroporation is more convenient for large-scale studies and allows transfer of both circular and linear DNA molecules. *In vivo* homologous recombination using phage functions, known as recombineering, which has been coupled with electrotransformation in *E*. *coli*^19^ and a few other bacteria^33–40^, is a powerful tool for *in vivo* genome editing. In *E*. *coli*, recombineering has enabled targeted multiplexed editing strategies such as MAGE (Multiplex Automated Genome Engineering)^53^, TRMR (Trackable Multiplex Recombineering)^54^ and CREATE (CRISPR EnAbled Trackable genome Engineering)^55^, all which take advantage of massive DNA delivery by electroporation, highlighting the importance of this transformation method when performing recombineering experiments.

The simple and highly efficient electrotransformation protocol reported in this study provides an opportunity to more easily manipulate *S*. *oneidensis*. Key features of this electroporation procedure are using small quantities of cells, making electrocompetent cells and performing electroporation at room temperature to avoid lysis and using cells at late exponential phase. With these conditions, ~4 × 10^6^ and ~5 × 10^3^ transformants/μg DNA were obtained using a non-methylated and *E*. *coli* methylated plasmid, respectively, thus even methylated plasmids can be used with our protocol. For simple plasmid transformation, late exponential phase cells produced the greatest increase in transformation efficiency, which was maintained when cells were frozen for long term storage at −80 °C. We were surprised by the high efficiency achieved under this condition, as this does not replicate across different bacteria^17,18,44^, although it is in agreement with *P*. *aureginosa* procedures^45^. We do not know why this difference in preferred growth phase prior to electroporation. We suspect it is not due simply to a higher input of viable cells, but possibly also to a reduced level of host nuclease activity in stationary phase.

In contrast to traditional genetic engineering strategies, recombineering allows researchers to rapidly and precisely introduce a variety of changes using linear DNA fragments. The use of ssDNA for recombineering, which was first described in *E*. *coli* over ten years ago^26^, is independent of a selective marker, does not leave any extraneous genetic scars and is mechanistically simpler than using dsDNA, requiring only the expression of the recombinase alone. However, the well-studied λ Red Beta and RecT recombinases were not functional in *S*. *oneidensis*. The number of recombinants obtained when expressing λ Red Beta or RecT was marginally above that of the recombinase-independent control. As shown in other bacteria^51^, *S*. *oneidensis* is able to recombine ssDNA in a recombinase-independent way at a frequency of ~10^−4^ recombinants/viable cells. According to Li and colleagues^56^, host factors such as replicative DNA polymerases and DNA ligase can play a role in completing recombination of the ssDNA at the replication fork^56^, which could explain why λ Red Beta and RecT are specific to *E*. *coli* and did not promote recombination in *S*. *oneidensis*.

In this study, we identified a λ Red Beta recombinase homolog in *Shewanella* sp. W3-18-1, termed W3 Beta, and demonstrated its functionality for ssDNA recombineering in *S*. *oneidensis*. We obtained a recombination efficiency of ~5%, similar to that of studies in *E*. *coli*^26^ and higher than previous λ Red Beta and RecT homologous systems found in other bacteria^33–40^. In addition, W3 Beta was also functional in *E*. *coli* and performed at similar efficiencies to λ Red Beta. The observation that W3 Beta outperforms other systems and that it is also functional in phylogenetically distant bacteria highlights the efficient recombineering activity of this recombinase.

Like other phage recombinases, W3 Beta appears to act at the replication fork since it displays a strand bias, as do similar systems^25,40^. We found that recombineering with W3 Beta results in higher efficiency when electrocompetent cells are prepared at room temperature instead of ice-cold temperature in *S*. *oneidensis* and *E*. *coli*, in agreement with our results using λ Red Beta in *E*. *coli*.

The recombineering system described here is the first effective and simple strategy for targeted and markerless genome-editing in *S*. *oneidensis*, yielding a high efficiency of recombinant formation that permits screening for desired mutations in the absence of selection. Our system was developed without strain engineering, however rational removal of single-strand exonucleases could potentially enhance efficiency as shown in *V*. *cholerae*^57^ and *E*. *coli*^49,56,58^. The cutting edge CRISPR/Cas-technology, which has been applied to diverse bacteria^59,60^, could be used as a powerful selection to enable facile isolation of recombinants. Efforts to further improve these methods are currently ongoing, including implementing a mechanism to cure plasmids from *S*. *oneidensis*. In its current state, our system should facilitate genome editing projects that require precise modifications of small to medium-throughput scale. We postulate that this study will pave the way to further strain engineering of *S*. *oneidensis*.

## Methods

### Bacterial Strains, plasmids and culture conditions

All strains and plasmids used in this study are listed in Table S1. *Shewanella* was routinely grown in LB liquid medium or on LB agar at 30 °C. When needed, culture media was supplemented with chloramphenicol (Cm, 6.5 µg/mL), kanamycin (Km, 50 µg/mL) or spectinomycin (Spect, 50 µg/mL). Plasmid pACYC’ was isolated from *E*. *coli* UQ950 and from methylation-minus *E*. *coli* GM1674, while plasmid pBTBX2 from UQ950 and methylation-minus *E*. *coli* GM2163.

Strain JG2150 was constructed by an in-frame insertion of *lacZ* from *E*. *coli* MG1655 under the native promoter of *mtrC* at the *glmS* site in *S*. *oneidesis* MR-1. In brief, 1 kb regions upstream and downstream of *glmS* were amplified and ligated into a suicide vector, pSMV3, which was transferred into MR-1 and screened for double recombination events, as previously described^10^.

### DNA manipulations

Plasmid DNAs were isolated using Invitrogen Plasmid mini Kit. The pACYC’ plasmid is a modified version of pACYC184^13^ harboring the chloramphenicol resistance gene (*cmR*); the tetracycline resistance gene (*tetR*) was removed by inserting the oriT RP4 in its place, since *tetR* shared 94 bp homology to another region in the plasmid backbone, the promoter of *cmR* was replaced by the promoter of *tetR* and, nucleotides 442–586 were removed due to 145 bp shared homology to the *E*. *coli* MG1655 genome.

All plasmids listed in Table S1 were created using Gibson Assembly Ultra Kit (Synthetic Genomics), except for removal of nucleotides 442–586 in pACYC184, which was performed by blunt ligation using NEB T4 DNA ligase. Primers listed in Table S3 were used to create linear dsDNA by PCR amplification in a 50 µL reaction with Q5 polymerase. The linear dsDNA vectors were DpnI digested for at least 2 hours and gel-purified. Inserts were amplified in a 50 µL reaction with Q5 polymerase and PCR purified. DNA fragments were assembled at a ratio 1/1 following supplier instructions and transferred in chemically competent *E*. *coli* UQ950. Cells were recovered for ~2 h in LB medium, plated on selective agar plates and incubated at 37 °C.

For colony PCR, a single colony was transferred to 25 µL dH_2_O, boiled at 95 °C for 5 min and frozen at −80 °C for 5 min. The cell debris was removed by centrifugation for 1 min and 2 µL of the supernatant were used as a source of template in a 20 µL reaction. GoTaq 2X MM (Promega) was used and supplier instructions were followed. After PCR, 10 µL aliquots were analyzed by agarose gel electrophoresis.

### Preparation of competent cells and electrotransformation

*Shewanella* cells were made competent by using a microcentrifuge-based procedure similar to that of *P*. *aureginosa*^45^. Overnight cultures were diluted to an OD_600_ of 0.08 and, once the cells reached an OD_600_ between 0.4–0.5 (~4 × 10^8^ CFU/ mL), three mL of cells were used per electroporation reaction. One mL was distributed per eppendorf tube and the cells were harvested at room temperature for one min at 7607 rcf. One mL of room temperature 10% glycerol was used to wash and combine three cell pellets into one. The cell pellets were washed two more times and suspended in the residual ~50 µL, which resulted on average 10^6^–10^7^ viable bacteria after electroporation. When overnight cultures were used, the washing steps were performed at 7607 rcf for two min. If competent cells were to be stored, cells were immediately frozen on liquid nitrogen and transferred to −80 °C. When using frozen cells, a 10 min defrost was allowed on ice followed by three min incubation with the plasmid DNA at room temperature prior to electroporation.

For electroporation, 250 ng of plasmid was mixed with ~50 µL of competent cells and the mixture was transferred to a room temperature 0.1 cm cuvette. After applying a pulse (settings: 10 µF, 600 Ω, 1.2 kV on a Bio Rad MicroPulser^TM^), one mL of room temperature liquid LB was added and transferred into a two mL Eppendorf tube for greater aeration. Cells were recovered at 30 °C shacking for two hours prior to plating on agar plates with antibiotics. The plates were incubated at 30 °C until colonies appeared. To determine number of viable cells, 10 µL of a range of 10-fold serial dilutions were spotted onto LB agar. To determine the number of transformants, 100 µL of the aliquots were plated on selective plates at appropriate dilutions to yield single colonies. For the lower efficiency conditions, the remaining cells (pelleted at 6,010 rcf for three min and suspended in remaining 100 µL LB) were also plated.

For the ice-chilled electrotransformation, competent cells were kept on ice for 15 min and then washed with cold 10% glycerol while keeping the tubes on ice. Electroporation was done with ice-chilled cuvettes.

### ssDNA oligo recombineering in *S*. *oneidensis*

All oligos described in Table S2, were ordered from IDT as 4 nmole ultramer dried DNA and suspended in dH_2_O to a concentration of 0.5 µg. Plasmids pX2SW3Beta, pX2λBeta and pX2RecT were transformed in host cells JG2150 or JG274, followed by plating on LB + Km and growth at 30 °C. The resulting strain cultured in LB + Km was supplemented with arabinose (20 mM) to express the recombinase to OD ≃ 0.4–0.5. Cells were made electrocompetent using sorbitol (1 M) as wash buffer and incubated with 2.5 µg (5 µL) of ssDNA for three min at room temperature prior to electroporation. After electroporation at 12 kV/cm, cells were allowed a 30 min (2 h for *rpsL* recombineering) recovery shaking at 30 °C prior to plating. For the experiments at lower temperatures, the same procedure as ice-chilled electrotransformation was followed after keeping the cells on ice for 15 or 60 min.

Following electroporation and the recovery period, aliquots were diluted 10^4^-fold on LB medium and all cells of this dilution were spread on LB + Km (50 µg/mL) and X-gal (60 µg/mL) agar plates to determine *lacZ*^−^ cells. To assess the frequency of *rpsL*K43R mutants, 100 µL of a range of 10-fold serial dilutions were plated on LB + Km (50 µg/mL) and Spect (50 µg/mL) agar plates at appropriate dilutions to yield single colonies, while viable cells were determined from colonies growing on LB + Km (50 µg/mL) plates. A reverse primer specific to the mutation introduced (lacZ-mut-R or rpsL-mut-R) was utilized for screening by colony PCR prior to sequencing.

## Supplementary information

Supplementary Information
