# Supplementary material for: A new recombineering system for precise genome-editing in Shewanella oneidensis strain MR-1 using single-stranded oligonucleotides
Source: Sci Rep. 2019 Jan 10;9:39. doi: 10.1038/s41598-018-37025-4 (PMC6328582; doi:10.1038/s41598-018-37025-4)
Supplement: Supplementary file 1 — Supplementary Information [file 41598_2018_37025_MOESM1_ESM.pdf]

SUPPLEMENTARY INFORMATION

A new recombineering system for precise genome-editing in *Shewanella oneidensis* strain MR-1 using single-stranded oligonucleotides

Anna D. Corts[1], Lynn C. Thomason[2], Ryan T. Gill[3] and Jeffrey A. Gralnick[1,*]

[1]BioTechnology Institute and Department of Plant and Microbial Biology, University of Minnesota-Twin Cities, St. Paul, MN 55108, USA, [2]RNA Biology Laboratory, Basic Science Program, Leidos Biomedical Inc., Frederick National Laboratory for Cancer Research, MD 21702, USA, [3]Department of Chemical and Biological Engineering, University of Colorado Boulder-Boulder, CO 80303, USA

*To whom correspondence should be addressed: gralnick@umn.edu

Inventory of Supplemental Information

Supplemental data

Figure S1. Comparison of electrotransformation at room temperature (RT) and at ice temperature (IT).
Figure S2. Effect of plasmid DNA methylation patterns on electroporation efficiency.
Figure S3. Effect of wash buffer in the number of viable cells after electroporation.
Figure S4. Effect of time frozen in 1 M sorbitol at -80 °C on *S. oneidensis* electrotransformation.
Figure S5. Effect of recombinase expression on *S. oneidensis* growth.
Figure S6. Recombinase protein alignments.
Figure S7. *S. oneidensis rpsL*K43R recombinants verification.
Figure S8. ssDNA recombineering in *E. coli* MG1655 by preparing electrocompetent cells at different temperatures.

Table S1. Strains and plasmids used in this work.
Table S2. Oligonucleotides used for chromosomal mutations.
Table S3. Primers used in this study.
Table S4. Gralnick Lab electroporation protocol for *S. oneidensis* MR-1**.**

Supplemental references

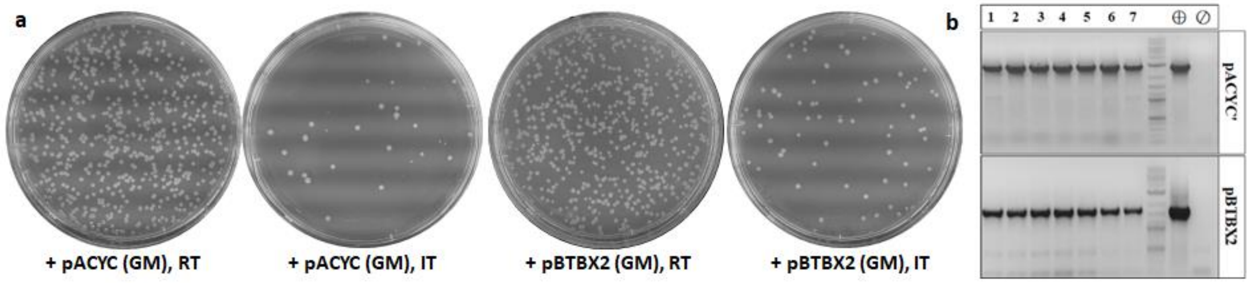

Figure S1. Comparison of electrotransformation at room temperature (RT) and ice temperature (IT). (**a**) Cell plates from transformation at RT and IT with each plasmid, pACYC' or pBTBX2, purified from methylation-minus *E. coli* GM1674 or GM2163 (GM), respectively. One mL of LB medium was added to the samples and the cell suspensions were recovered at 30 °C for 2 h. After the recovery step, aliquots of cells were not diluted but 900 µL of cells (pelleted and suspended in remaining 100 µL LB) were plated on selective agar plates. (**b**) Seven transformants from (a) at RT transformation with each plasmid were analyzed by colony PCR, which revealed the presence of the plasmid as expected. ⊕ indicates a positive plasmid control, ∅ indicates a negative control *S. oneidensis* with no plasmid. The gel was cropped to show the relevant information and no high-contrast of the image was used.

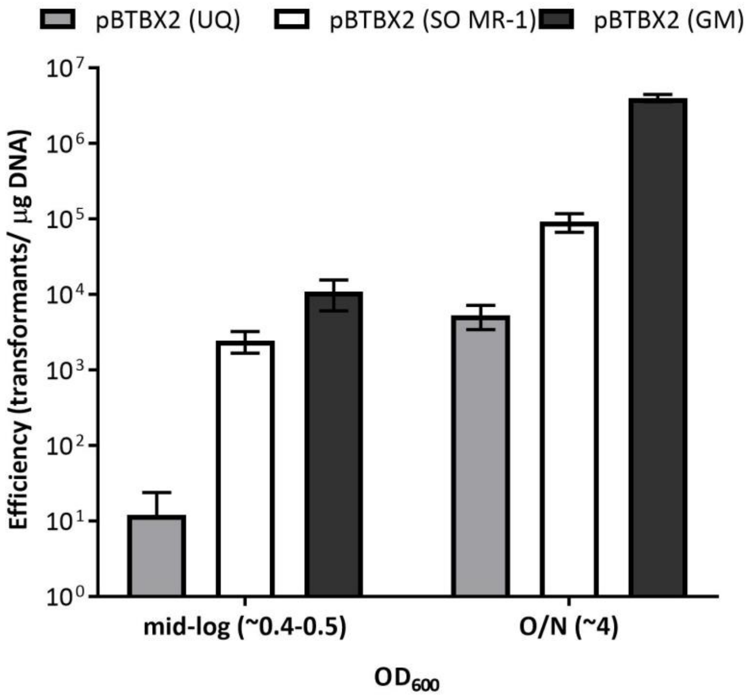

Figure S2. Effect of plasmid DNA methylation patterns on electroporation efficiency. 250 ng of pBTBX2 plasmid DNA purified from methylation-proficient *E. coli* UQ950*,* methylation-minus *E. coli* GM2163 or *S. oneidensis* MR-1 was used for electroporation. One mL of LB medium was added to the samples and the cell suspensions were recovered at 30 °C for 2 h. Aliquots were then plated on LB + Km plates at appropriate dilutions to yield single colonies. Transformants were counted after ~36 h incubation at 30 °C. The error bars represent standard error from three independent experiments.

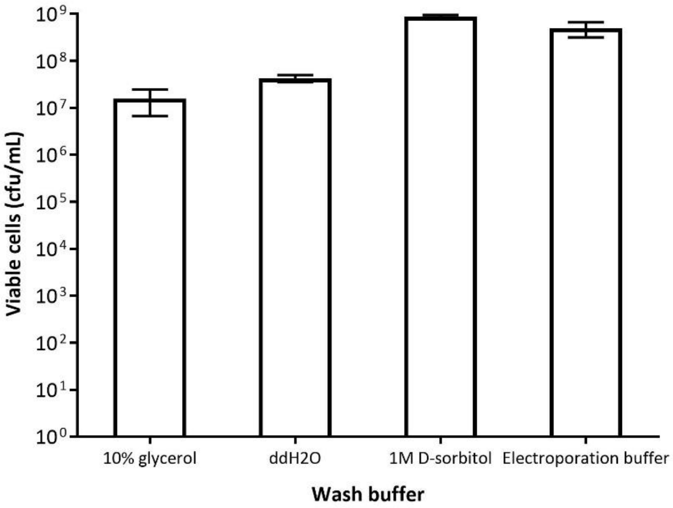

Figure S3. Effect of wash buffer on the number of viable cells after electroporation. 10% glycerol, ddH_2O, 1 M D-sorbitol or electroporation buffer[1] was used. 250 ng of pBTBX2 plasmid DNA from methylation-minus *E. coli* GM2163 was transferred by electroporation. One mL of LB medium was added to the samples and the cell suspensions were recovered at 30 °C for 2 h. 10 µL of a range of 10-fold serial dilutions were spotted onto LB agar and colonies were counted after ~24 h incubation at 30 °C. The error bars represent standard error from three independent experiments.

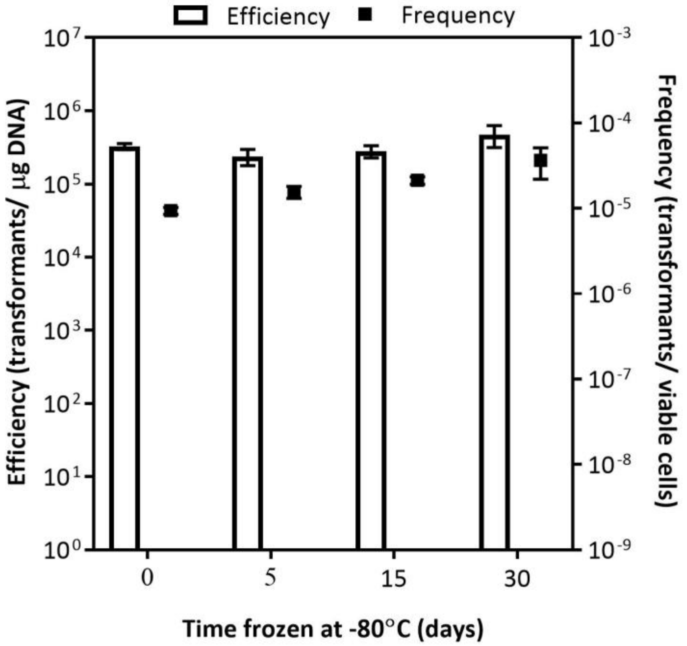

Figure S4. Effect of time frozen in 1 M sorbitol at -80 °C on *S. oneidensis* electrotransformation. One mL of overnight culture was washed with 1 M sorbitol as described in the Methods section and frozen immediately. At the times specified, cells were tested for electrotransformation with 250 ng of pBTBX2 plasmid DNA isolated from the methylation-minus *E. coli* strain GM2163. One mL of LB medium was added to the samples and the cell suspensions were recovered at 30 °C for 2 h. Aliquots were plated on LB + Km plates at dilutions to yield single colonies. Transformants were counted after ~36 h incubation at 30 °C. The error bars represent standard error from three independent experiments.

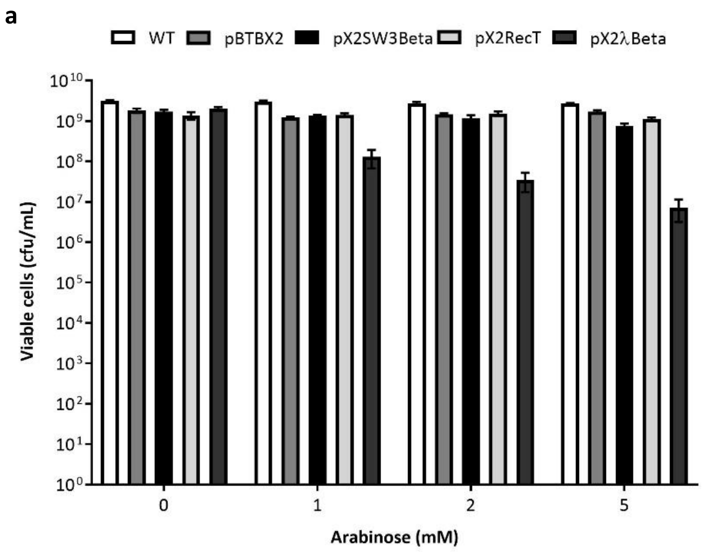

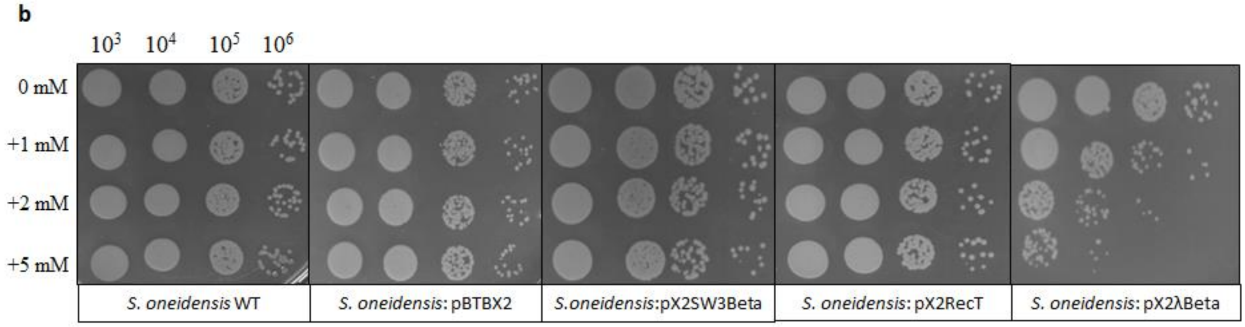

Figure S5. Effect of recombinase expression on *S. oneidensis* growth. (**a**) Effect of varying arabinose concentration on viability of wild-type cells (WT), cells carrying the empty vector pBTBX2 and cells with the vector expressing the recombinase under pBAD (W3 Beta, RecT or λ Red Beta). (**b**) Colony forming units from a range of 10-fold serial dilutions. Cells were subcultured to a starting OD_{600} of 0.08 and grown to an OD_{600} of ~0.4-0.5 and then, the specified arabinose concentration was added. Cells were induced for 3 h and after induction, aliquots were spotted onto LB agar (WT cells) or LB + Km (cells with plasmid) at different dilutions. Colonies were counted after ~24 h incubation at 30 °C. The error bars represent standard error from three independent experiments.

a

```
W3 Beta 6    LIQRFAERF---SVDPNKLFDTLKATAFKQRDGSAPTNEQMMALLVVADQYGLNPFTKEI   62
λ Beta  8    LAGKLAERVGMDSVDPQELITTLRQTAFK---GDA-SDAQFIALLIVANQYGLNPWTKEI   63

W3 Beta 63   FAFPDKQAGIIPVVGVDGWSRIINQHDQFDGMEFKTSENKVSLDGAKECPEWMECIIYRR  122
λ Beta  64   YAFPDKQNGIVPVVGVDGWSRIINENQQFDGMDFEQDNESCT------------CRIYRK  111

W3 Beta 123  DRSHPVKITEYLDEVYRPPFEGNGKNGPYRVDGPWQTHTKRMLRHKSMIQCSRIAFGFVG  182
λ Beta  112  DRNHPICVTEWMDECRREPFK--TREG-REITGPWQSHPKRMLRHKAMIQCARLAFGFAG  168

W3 Beta 183  IFDQDEAERIIEGQA   197
λ Beta  169  IYDKDEAERIVENTA   183
```

b

```
W3 Beta 183  DGPWQTHTKRMLRHKSM   170
RecT    206  NGPWVTHWEEMAKKTAI   222

W3 Beta 183  PVVGVDGWSRIINQHDQFDGMEFKTSENKVSLDGAKECPEWM   115
RecT    169  PVTHVYAVARLKDGGTQFEVMTRKQIELVRSLSKAGNNGPWV   210

W3 Beta 183  RHKSMIQCSRIA   177
RecT    48   RHMTAERMIRIA   59
```

Figure S6. Recombinase protein alignments. The National Center Biotechnology Institute Blastp tool was used. Yellow highlighting indicates exact identity, whereas green indicates similarity. (**a**) W3 Beta versus λ Red Beta. (**b**) W3 Beta versus RecT.

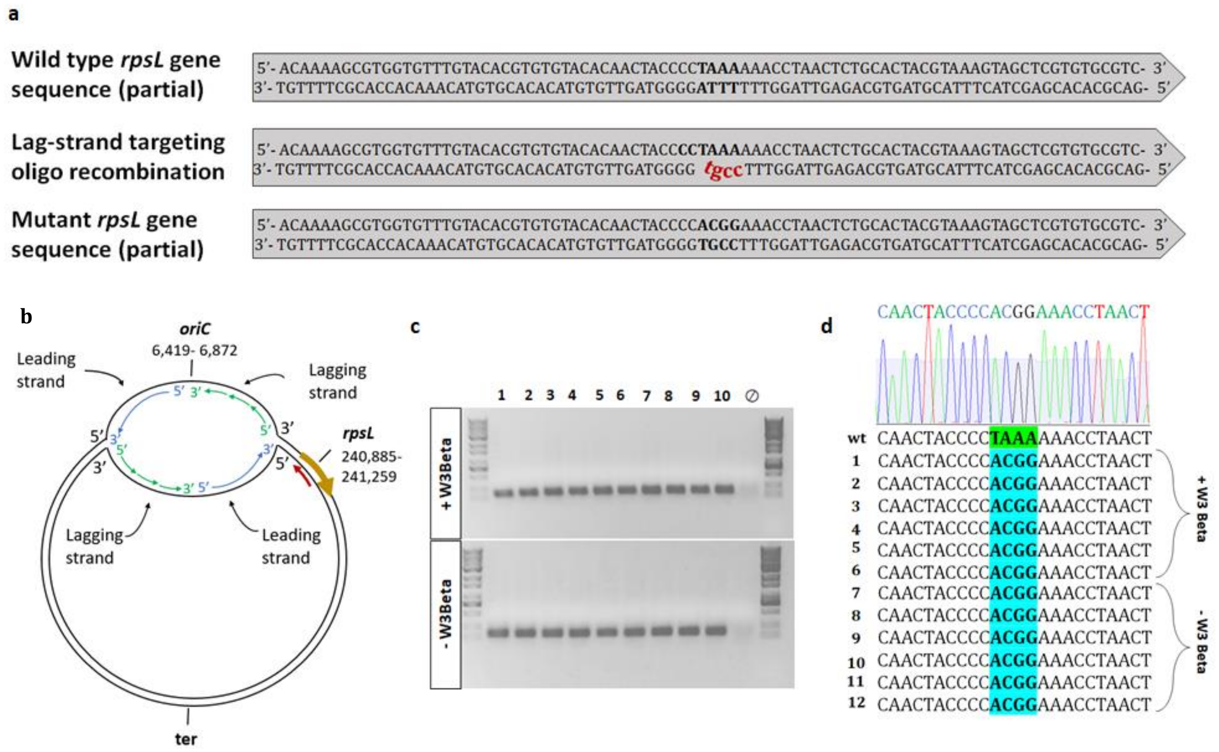

Figure S7. *S. oneidensis rpsL*K43R recombinants verification. (**a**) Mutagenesis of chromosomal *rpsL* in *S. oneidensis*. Sequence corresponding to the site of the mutation is shown in bold. A recombinogenic oligonucleotide (oligo) targeting the lagging strand was utilized to introduce four consecutive base pair changes, which resulted in the K43R mutation and a synonymous mutation in P42, shown in red. (**b**) Organization of the *S. oneidensis* MR-1 chromosome showing the location of *rpsL* respective to the origin of replication (*oriC*). The relative position of *oriC* (determined using DoriC database) and terminus (estimated as the location of the origin plus half of the chromosome length) define two replichores through which DNA synthesis proceeds. As the oligos mimic Okazaki fragments at the replication fork, the orientation of the target gene in respect to the *oriC* needs to be known beforehand for an optimal recombineering efficiency. Shown in red is the 5'→ 3' alignment of the recombinogenic oligo, complement to the lagging strand, to target *rpsL*. (**c**) Mutants were identified as streptomycin resistant (StepR) colonies. Ten mutants from each experiment, the + W3 Beta and – W3 Beta (pBTBX2 empty plasmid control), were screened by colony PCR with a reverse primer specific to the DNA change introduced, validating the correct mutation in all colonies. ∅ indicates a PCR negative control of wild-type *rpsL*. The gel was cropped to show the relevant information and no high-contrast of the image was used. (**d**) Sequence confirmation of 6 recombinants identified by StrepR from each experiment, which further revealed the mutation of the DNA sequence TAAA to ACGG for all samples, as expected.

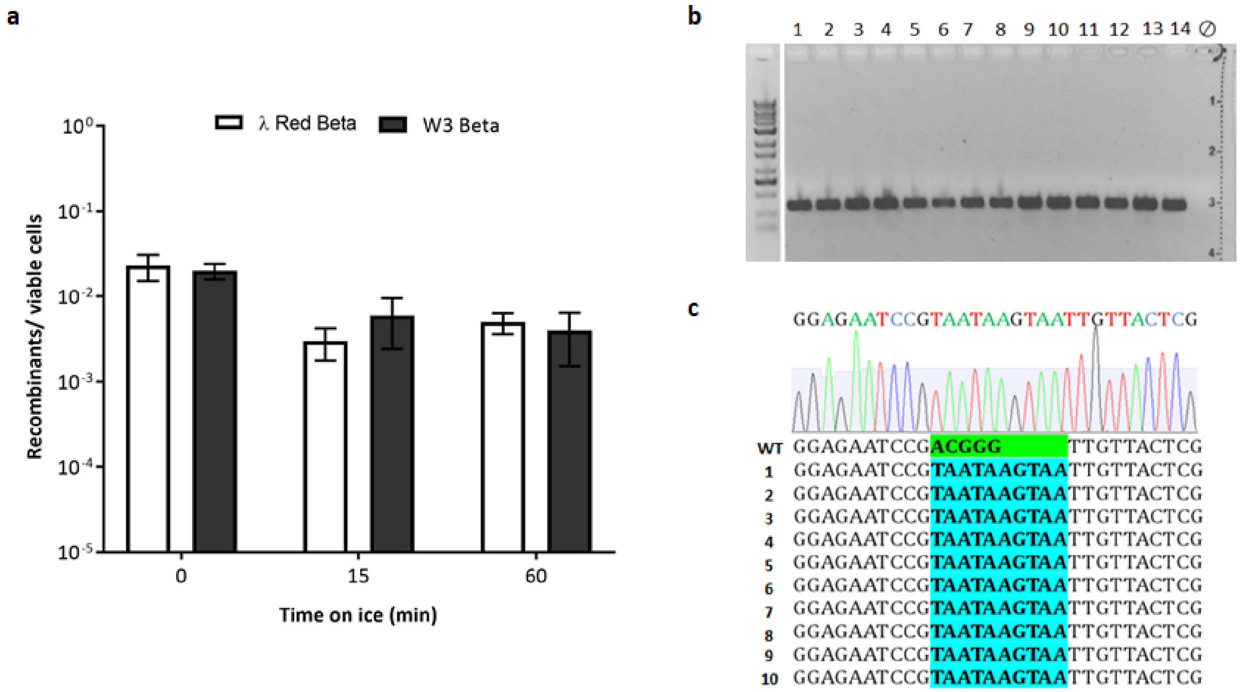

Figure S8. ssDNA recombineering in *E. coli* MG1655 by preparing electrocompetent cells at different temperatures. (**a**) Comparison of λ Red Beta and W3 Beta activity in ssDNA recombineering using a lag-strand targeting oligo with 80nt homology arms to disrupt *lacZ,* as shown in Fig. 4a. The number of recombinants was calculated by scoring blue colonies containing white sectors on the LB + Km + X-gal agar plates. The error bars represent standard error from three independent experiments. (**b**) Sectored colonies were streaked for isolated *lacZ*$^-$ pure colonies and screened by colony PCR with a reverse primer specific to the DNA change introduced, validating the correct mutation in all colonies. ∅ indicates a PCR negative control of wild-type *lacZ*. The gel was cropped to show the relevant information and no high-contrast of the image was used. The molecular size marker was run on the same gel as the samples shown. The white line indicates the gel contained other samples not relevant. (**c**) Sequence confirmation of 10 recombinants identified by white/blue screening, which revealed the mutation of the DNA sequence ACGGG to TAATAAGTAA for all samples, as expected.

Table S1. Strains and plasmids used in this work.

Strain or plasmid	Description	Reference or source
**Strain**		
JG274	*S. oneidensis* MR-1, wild type	Gralnick Lab
JG239	*Shewanella sp.* W3-18-1	Gralnick Lab
JG2150	*S. oneidensis* MR-1, *lacZ*	Gralnick Lab
JG3653	*S. oneidensis* MR-1, pBTBX2	This study
JG4123	*S. oneidensis* MR-1, pX2SW3Beta	This study
JG3909	*S. oneidensis* MR-1, pX2λBeta	This study
JG3916	*S. oneidensis* MR-1, pX2RecT	This study
JG4140	*S. oneidensis* MR-1, *lacZ,* pBTBX2	This study
JG4127	*S. oneidensis* MR-1, *lacZ,* pX2SW3Beta	This study
JG3940	*S. oneidensis* MR-1, *lacZ,* pX2λBeta	This study
JG3942	*S. oneidensis* MR-1, *lacZ,* pX2RecT	This study
UQ950	*E. coli* methylation-proficient	[2]
GM2163	*E. coli* methylation-minus, CmR	CGSC#: 6581
GM1674	*E. coli* methylation-minus	CGSC#7971
JG4130	*E. coli* MG1655, pX2SW3Beta	This study
JG4128	*E. coli* MG1655, pX2λBeta	This study
**Plasmid**		
pACYC'	Modified pACYC184, p15a ori, CmR	[3]
pBTBX2	pBBR1 ori, KmR, pBAD	[4]
pX2SW3Beta	pBBR1 ori, KmR, pBAD, W3Beta	This study
pX2λBeta	pBBR1 ori, KmR, pBAD, λ Red Beta	This study
pX2RecT	pBBR1 ori, KmR, pBAD, RecT	This study

Table S2. Oligonucleotides used for chromosomal mutations.

Name	Sequence (5'-> 3')
80nt-lag-*lacZ*	**5'**acggttacgatgcgcccatctacaccaacgtgacctatcccattacggtcaatccgccgtttgttcccacggagaatccgTAATAAGTAAttgttactcgctcacatttaatgttgatgaaagctggctacaggaaggccagacgcgaattatttttgatggcgttaact
80nt- lead-*lacZ*	**5'**agttaacgccatcaaaaataattcgcgtctggccttcctgtagccagctttcatcaacattaaatgtgagcgagtaacaaTTACTTATTAcggattctccgtgggaacaaacggcggattgaccgtaatgggataggtcacgttggtgtagatgggcgcatcgtaaccgt
50nt- lag- *lacZ*	**5'**tgacctatcccattacggtcaatccgccgtttgttcccacggagaatccgTAATAAGTAAttgttactcgctcacatttaatgttgatgaaagctggctacaggaaggcc
50nt- lead-*lacZ*	**5'**ggccttcctgtagccagctttcatcaacattaaatgtgagcgagtaacaaTTACTTATTAcggattctccgtgggaacaaacggcggattgaccgtaatgggataggtca
40nt- lag-lacZ	**5'**cattacggtcaatccgccgtttgttcccacggagaatccgTAATAAGTAAttgttactcgctcacatttaatgttgatgaaagctggcta
40nt- lead-*lacZ*	**5'**tagccagctttcatcaacattaaatgtgagcgagtaacaaTTACTTATTAcggattctccgtgggaacaaacggcggattgaccgtaatg
40nt- lag-*rpsL*	**5'**gacgcacacgagctactttacgtagtgcagagttaggtttCCGTggggtagttgtgtacacacgtgtacaaacaccacgcttttgt
40nt- lead-*rpsL*	**5'**acaaaagcgtggtgtttgtacacgtgtgtacacaactaccccACGGaaacctaactctgcactacgtaaagtagctcgtgtgcgtc

Table S3. Primers used in this study. Lower case designates Gibson assembly overhangs.

**Name**	**Sequence (5'-> 3')**
**Primers for plasmid modification of pACYC184:**	
pACYC-R	TATGGGGCTGACTTCAGGTGC
pACYC-F	TAACGGATTCACCACTCCAAG
oriT4pACYC-F	gcacctgaagtcagccccataTGTAGACTTTCCTTGGTGTATCCAACG
oriT4pACYC-R	cttggagtggtgaatccgttaGAATAAGGGACAGTGAAGAAGGAACAC
tet-prom-F	caacttttggcgaaaatgagacgtCGATATAAGTTGTAATTCTCATGTTTGACAGCTT
tet-prom-R	tggtatatccagtgatttttttctccatACACGGTGCCTGACTGC
pACYC-tetpr-F	ATGGAGAAAAAAATCACTGGATATACCA
pACYC-tetpr-R	ACGTCTCATTTTCGCCAAAAGTTG
pACYC-Rem-F	gcgaagtgatcttccgtcacagGGCTTACTATGTTGGCACTGATGAG
pACYC-Rem-R	ctcatcagtgccaacatagtaagccCTGTGACGGAAGATCACTTCGC
pACYC-lig-F	GGCTTACTATGTTGGCACTGATGAG
pACYC-lig-R	CTGTGACGGAAGATCACTTCGC
**Primers for construction of recombineering plasmids:**	
pX2-R	GGGTATATCTCCTTCTTAAAGTTCGTATC
pX2-F	TCTAGAGAATTCGTCAACGA
LBeta4X2-F	cgaactttaagaaggagatatacccATGAGTACTGCACTCGCAACG
LBeta4X2-R	cgttgacgaattctctagaTCATGCTGCCACCTTCTGCT
RecT4X2-F	cgaactttaagaaggagatatacccATGACTAAGCAACCACCAATC
RecT4X2-R	cgttgacgaattctctagaTTATTCCTCTGAATTATCGATTACACTG
W3Bet4X2-F	cgaactttaagaaggagatatacccATGGAAAAACCAAAGCTAATCCAACG
W3Bet4X2-R	cgttgacgaattctctagaCTAAGAAGCTAAAGGCTGTGTGAGCG
**Primers for plasmid sequence verification:**	
end-KmR-F	ATACCAGGATCTTGCCATCC
araC-F1	TATGGAACTGCCTCGGTGAG
araC-F2	GACGAAAGTAAACCCACTGG
pBAD-F	ATTAGCGGATCCTACCTGACG
LBet-F	CGCATCATCAATGAAAACCAGCAG
W3Bet-F	ATCCATGATCCAGTGTTCCCGC
pBTBX2-R2	AATCCCTGTGGTCAAGCTCG
**Primers for plasmid analysis:**	
P1	GTTTCACTTGATGCTCGATGAG
P2	AATCCCTGTGGTCAAGCTCG
P3	CAGGCATTTGAGAAGCACACGG
P4	GCGATGCAGATCCGGAACAT
**Primers for screening recombinants:**	
lacZ-F	TGGCGTAATAGCGAAGAGG
lacZ-R	CCTGATCTTCCAGATAACTGCC
lacZ-mut-R	GTGAGCGAGTAACAATTACTTATTA
rpsL-F	ATGGCAACTGTAAACCAG
rpsL-R	TTAAGACTTAGGACGCTTAGC
rpsL-mut-R	CAGAGTTAGGTTTCCGTGG

Table S4. Gralnick Lab electroporation protocol for *S. oneidensis* MR-1.

STEP	DESCRIPTION	NOTES
**1. Preliminary Steps**	a) Culture a colony overnight in LB liquid medium at 30 °C with shaking (~200 rpm)<br>b) Make 10% v/v glycerol<br>c) Obtain purified plasmid DNA	* DNA concentration should be ~100 ng/µL. Use a Nanodrop if available; if 260/230 and 280/260 ratios are not ~2 and ~1.8, respectively, the efficiency may be lower.
**2. Making electrocompetent cells at room temperature:**	a) Prepare ~100 ng plasmid DNA in a 2 mL Eppendorf tube. Perform a control with no DNA<br>b) Collect 1 mL of an overnight culture in an Eppendorf tube, spin at 7,906 rcf for 1 min<br>c) Wash cells three times with 1 mL 10% glycerol, spinning at 7,906 rcf for 2 min<br>d) After the third wash, decant the glycerol and leave ~70 µL for suspending the cells<br>e) Suspend cells gently by shaking the tube and pipetting with P100, then mix cells with the DNA by swirling around with the tip	*When suspending cells with glycerol, be gentle pipetting with P1000 ~10 times.<br>* When decanting the glycerol after spins, use care to not lose the pellet; gently tap the tube 3 times.<br>* If desired, freeze cells right away in liquid nitrogen and transfer to -80 °C.<br>*A 0.1 cm cuvette fits ~100 µL max. A 0.2 cm cuvette requires higher voltages.
**3. Performing electroporation at room temperature:**	a) Transfer the mixture immediately into a 0.1 cm cuvette without making bubbles<br>b) Electroporate at 1.2 kV. Time constant (tc) should be ~5<br>c) Quickly add 1 mL LB into the cuvette and mix gently while pipetting up and down<br>d) Transfer the cells into the same 2 mL tube you had the DNA in<br>e) Let the cells recover at 30 °C for 2 h with shaking ~200 rpm	* If using frozen cells, defrost on ice ~10 min, then incubate for 3 min with the DNA at room temperature to allow transient swelling of cells.<br>* Electroporator settings: 10 µF, 600 Ω (if different, voltage may need to be optimized).<br>* If tc is not ~5, efficiency may be lower.
**4. Plating:**	a) Make 10x serial dilutions in LB<br>b) Plate several different dilutions<br>c) Incubate plates at 30 °C for ~24-36 h until colonies appear	* Depending on the source of the DNA, efficiency varies (see Fig. S2 as a reference to know which diluted aliquots to plate).<br>* No colonies should appear on the control with no DNA (if the cells are not diluted enough, false positives can appear on a lawn of dead cells, depending on the antibiotic used).

Supplemental references

1. Weinstock, M. T., Hesek, E. D., Wilson, C. M. & Gibson, D. G. *Vibrio natriegens* as a fast-growing host for molecular biology. *Nat. Methods* **13,** 849–851 (2016).
2. Saltikov, C. W. & Newman, D. K. Genetic identification of a respiratory arsenate reductase. *Proc. Natl. Acad. Sci.* **100,** 10983–10988 (2003).
3. Myers, C. R. & Myers, J. M. Replication of plasmids with the p15A origin in *Shewanella putrefaciens* MR-1. *Lett. Appl. Microbiol.* **24,** 221–5 (1997).
4. Prior, J. E., Lynch, M. D. & Gill, R. T. Broad-host-range vectors for protein expression across gram negative hosts. *Biotechnol. Bioeng.* **106,** 326–332 (2010).
